# Interaction between genetic predisposition to successful ageing and chronic air pollution on lung disease in elderly women: results of the German SALIA cohort

**DOI:** 10.1136/bmjresp-2025-003226

**Published:** 2025-11-04

**Authors:** Sara Kress, Michael Lau, Claudia Wigmann, Michael J Abramson, Holger Schwender, Tamara Schikowski

**Affiliations:** 1 IUF - Leibniz Research Institute for Environmental Medicine, Düsseldorf, Germany; 2 Mathematical Institute, Faculty of Mathematics and Natural Sciences, Heinrich Heine University Düsseldorf, Düsseldorf, Germany; 3 School of Public Health and Preventive Medicine, Monash University, Melbourne, Victoria, Australia; 4 School of Public Health, Bielefeld University, Bielefeld, Germany

**Keywords:** Pulmonary Disease, Chronic Obstructive, Machine Learning

## Abstract

**Objective:**

To investigate the interplay between the genetic predisposition to successful ageing and air pollution on lung disease in healthy aged German women under the hypothesis that ageing and lung diseases share mechanisms of oxidative stress and inflammation that can be regulated by genetic predisposition and environmental factors.

**Design:**

German Study on the influence of Air pollution on Lung function, Inflammation and Aging prospective cohort between baseline (1985–1994) and follow-up (2007–2010).

**Setting:**

Urban Ruhr area and the adjacent rural Münsterland in Germany.

**Participants:**

At baseline, 4874 women aged 55 years living between 1985 and 1994 in the setting and at follow-up examination, 834 of them participated.

**Main outcome measures:**

Chronic lung disease was defined as any of asthma, chronic bronchitis, cough (with sputum) or chronic obstructive pulmonary disease. Chronic individual exposures to nitrogen dioxide (NO_2_), nitrogen oxides, particulate matter with median aerodynamic diameters <2.5 (PM_2.5_), PM_10_, PM_coarse_ and PM_2.5 absorbance_ based on European Study of Cohorts for Air Pollution Effects land-use regression models were used. Main and interaction effects between the genetic risk score (77 single-nucleotide polymorphisms (SNPs) related to successful ageing) and air pollutant exposures were investigated using adjusted logistic regression models.

**Results:**

In 560 women (67–80 years), chronic lung disease was present in 156. Higher exposure to air pollution was associated with increased odds by up to 43% per IQR-increase in NO_2_ (IQR=11.6 µg/m³, 95% CI 1.15 to 1.77). The genetic make-up reduced the negative impact of air pollution (gene–environment interaction with NO_2_: OR=0.66, 95% CI 0.45 to 0.96), while a healthy lifestyle further strengthens this association.

**Conclusions:**

In elderly women, genetic predisposition based on successful ageing SNPs likely reduces the negative impact of air pollution on chronic lung disease, while a healthy lifestyle further strengthens this association.

**WHAT IS ALREADY KNOWN ON THIS TOPIC**

- The interplay between air pollution exposure and individual genetic susceptibility affects respiratory health.
- Underlying oxidative stress and inflammatory pathways are also involved in the ageing process.
- There is limited research on lung disease in the elderly, particularly including the genetic predisposition to successful ageing.

**WHAT THIS STUDY ADDS**

- Our study suggests that a higher exposure to air pollutants increases the odds of chronic lung disease in older ages.
- We found that a genetic predisposition based on successful ageing genetic variants likely reduces the negative impact of air pollution on chronic lung disease in elderly women.
- This association was further strengthened in women with a healthy lifestyle.

**HOW THIS STUDY MIGHT AFFECT RESEARCH, PRACTICE OR POLICY**

- Further studies should take a broader perspective on ageing and chronic diseases in the elderly. Combining the evidence of both fields could help to better understand the ageing process and chronic diseases.
- Our findings emphasise the importance of a healthy environment, including reduction of air pollution and having a healthy individual lifestyle in the ageing population as two modifiable public health targets.

## Introduction

 Chronic respiratory diseases are likely linked through oxidative stress and inflammatory pathways to air pollution exposure, whereby there might be an interaction with the individual genetic susceptibility^⁠^.^1 2^ Interestingly, underlying oxidative stress responses, including inflammatory mechanisms, are also likely involved in the ageing process^⁠^.^3^,^6^ The process of lung ageing associated with environmental exposures is regulated by oxidative stress, inflammation and genetic changes or damage^⁠^.^7^

So far, studies of centenarians have indicated that successful ageing, described as the avoidance of chronic diseases in the elderly^⁠^,^1 8 9^ is a complex construct comprising various genetic interplays, lifestyle and gene–environment interactions (GxE)^⁠^.^3 4 6 10^ Candidate genes that are linked to successful ageing, as well as specific alleles in healthy elderly that may buffer risk effects, have already been identified^⁠^.^611^,^13^ To the best of our knowledge, only one observational study has investigated the association between genes relevant to successful ageing and respiratory health, which showed that a higher genetic risk score (GRS) for lifespan was associated with fewer respiratory diseases.^13^

In the context of an ageing global population, it is of interest to identify preventative mechanisms for age-related diseases to achieve successful ageing. However, epidemiological studies have generally lacked sufficient focus on the population of healthy older adults. Furthermore, existing GxE studies of respiratory health in adults or the elderly have included genetic variants related to the respiratory health outcome only.^14 15^

The present study contributes to examining whether an individual’s genetic predisposition to successful ageing can elucidate mechanisms of lung ageing in an environmental context. Therefore, we aim to investigate the interaction between polygenic susceptibility based on successful ageing-related single-nucleotide polymorphisms (SNPs) and air pollution exposure on chronic lung disease in a cohort of healthy aged German women. It is hypothesised that, when air pollution exposure is considered, a genetic predisposition based on successful ageing SNPs reduces the risk of chronic respiratory diseases in older age.

## Methods

### Study population

The Study on the influence of Air pollution on Lung function, Inflammation and Aging (SALIA) is an ongoing cohort that included 4874 women aged 55 years between the years 1985 and 1994 living in the urban Ruhr area and the adjacent rural Münsterland in Germany. The SALIA study was initiated by the Government of North Rhine-Westphalia as part of the Clean Air Plan. The highly industrialised Ruhr district was chosen to represent pollution from the steel and coal industries and high-traffic areas, with non-industrial reference areas. To avoid bias related to occupational exposure from working in the steel industry or coal mining, men were excluded. Details have been described before.^16 17^ In this analysis, data were used from the baseline, first (year 2006) and second follow-up examinations (2007–2010).

### Assessment of chronic lung disease

Chronic lung disease was defined as self-responses of any of the following conditions: asthma, chronic bronchitis, cough (with sputum) or chronic obstructive pulmonary disease ever diagnosed by a physician at the second follow-up examination (mean age of 74 years).

### Assessment of air pollution exposure

Individual exposures to the air pollutants nitrogen dioxide (NO_2_), nitrogen oxides (NO_x_), particulate matter with median aerodynamic diameters <2.5 (PM_2.5_), <10 (PM_10_), 2.5–10 µm (PM_coarse_) and the reflectance of PM_2.5_ filters which is an indicator of black carbon (PM_2.5 absorbance_) were estimated. Within the European Study of Cohorts for Air Pollution Effects (ESCAPE),^18 19^ 14-day measurements were conducted of PM_2.5/10/2.5 absorbance_ from 20 monitoring sites in each season (cold, warm or intermediate temperatures from October 2008 to November 2009). NO_2_ was monitored at 40 sites. The concentrations of PM_coarse_ were calculated by subtracting PM_2.5_ from PM_10_. The values were adjusted for the true long-term average using continuously central monitoring site measurements for a complete year. Land-use regression models predicted the concentrations at the individual home addresses per each examination.

To model chronic air pollution exposure of about 15 years before the respiratory assessments, we averaged the annual mean exposures at the baseline and first follow-up examinations. Higher concentrations of air pollution represented higher exposure and were standardised in IQR.

### Assessment of genetic variants and calculation of the GRS

Genome-wide genotyping using Axiom Precision Medicine Research Array (Affymetrix, Santa Clara, California, USA) (GRCh37/hg19) was performed in 752 blood or saliva samples. We performed quality controls^20^ and genotype imputation against the Haplotype Reference Consortium using the Michigan Imputation Server.^21^ After postimputation quality control, 586 individuals and 7 643 653 SNPs remained. Details on the quality controls and imputation have been described before.^22^

Based on candidate genes and genome-wide association studies (GWAS) on ageing phenotypes, 89 SNPs were identified, of which 77 were available in the SALIA cohort (online supplemental table S1).

GRS precisely estimates individual genetic susceptibility concerning the biological plausibility and statistical robustness,^23^ while summarising the individual number of risk alleles of selected SNPs.^24^

The GRS was defined as an internally constructed function that assigns the alleles of the 77 SNPs a risk estimate. The GRS was constructed as proposed by Lau *et al*^25^ using random forests,^26^ which train ensembles of decision trees using ‘out-of-bag’ predictions. In general, random forest models have the advantage compared with traditional regression models to model complex, non-linear relationships and provide robust predictions for data containing noise. The advantage of the use of bagging (bootstrap aggregating), an ensemble learning technique in machine learning, is that the full dataset can be used for both GRS construction and subsequent GxE interaction testing without overfitting. A higher GRS represented a higher individual risk profile for chronic lung disease based on risk-increasing (ageing-related) alleles and was standardised in IQRs.

### Assessment of potential confounders

Potential confounders were selected a priori based on the current literature. The ageing process involves determinants including sex^⁠^,^4^,^627^ socioeconomic settings^⁠^,^4 6 28^ educational level,^29^ obesity^⁠^,^5 6 29^ exercise^⁠^,^5 6^ smoking^⁠^,^7 29^ temperature^⁠^^6^ and urban–rural environment.^30^

A multicentre cohort study on chronic air pollution exposure and adult lung function included the following potential confounders: age, height, sex, body mass index (BMI), educational level and smoking status.^31^

In this study, we included the following covariates: age, BMI, educational level (low as the reference, medium, high), smoking status (never as the reference, ever), passive smoking (never as the reference, ever), indoor air pollution (dampness, mould or cooking with gas at home) and residential moving in the observation time.

### Statistical analysis

Descriptive statistics were used to summarise individual characteristics, chronic lung disease and air pollution exposures. Using R V.4.1.2,^32^ crude and adjusted multivariable logistic regression models based on complete cases were fitted to chronic lung disease with each air pollutant separately to avoid collinearity between pollutants. We investigated the genetic main effect (per IQR increase), environmental main effect (per IQR increase) and the GxE effect via a multiplicative interaction term. The results are presented in ORs, 95% CIs and p values.

To test the robustness of the adjusted model with GRS constructed using random forests (main model), we repeated the GxE analysis using an established approach to construct internal weighted GRS.^33^ We used a 50:50 split of the data into a training and test dataset, which is generally recommended for testing the association between a GRS and an outcome and has been determined to be an effective allocation for GxE interaction studies in particular.^33^,^35^ The training dataset was used to calculate the weight of each SNP using the marginal associations between the SNPs and the outcome in a regularised classification method, the elastic net logistic regression, and the test dataset to calculate the individual GRS and perform the GxE analysis.^33^,^35^

Additionally, we interpreted the main GxE model building two contingency tables with relative risks of low versus high GRS and low versus high air pollution resulting in four subgroups as proposed by Ottman.^36^ GRS was divided by the median into high-risk versus low-risk GRS and exposure to air pollution was: (1) divided by the median to be consistent with the GRS and specific to the study sample and (2) exceeding versus not exceeding the European Union (EU) thresholds on ambient air quality and cleaner air for Europe^37^ to interpret the results in accordance with the official guidelines and enhance the interpretability for policies.

We performed stratified GxE analyses according to (1) individuals with healthy vs unhealthy individual lifestyle (BMI ≥30 kg/m², ever smoking, 0 hours of sport per week or walking/riding less than 15 min per week) and (2) individuals with healthy vs unhealthy housing conditions such as indoor air pollution including dampness, mould, cooking with gas or passive smoking at home.

### Patient and public involvement

Since the baseline observation of the SALIA participants, the women can provide feedback on questionnaires and general aspects. The SALIA participants were not involved in the conception and design of this specific study. There were no fundings and time allocated to public involvement, so we were unable to involve them in our study conception.

## Results

The second follow-up examination of the SALIA study included 834 women. In this analysis, 560 women with genetic information (67–80 years old, on average slightly overweight) were included (table 1). Chronic lung disease was present in 156 women. The median chronic air pollution exposure was close to the annual limits for NO_2_, PM_2.5_ and PM_10_ of the EU (RL 2008/50/EG).^37^ However, compared with the recent air quality guidelines of the WHO, all individuals were exposed to higher air pollution exposure than recommended.^38^

**Table 1:** Description of the study individuals, chronic lung disease and air pollution exposures

	SALIA
N	560
Chronic lung disease No. (%)	156 (27.9)
Mean age (years)±SD	73.5±3.0
Mean body mass index (kg/m²)±SD	27.3±4.3
Educational level of the participant or spouse No. (%)	
Less than 10 years	96 (17.2)
10 years	279 (49.9)
More than 10 years	184 (32.9)
Smoking No. (%)	
Ever smoker	104 (18.6)
Passive smoking	341 (61.2)
Indoor air pollution No. (%)*	138 (24.6)
Residential move No. (%)	71 (12.7)
Healthy individual lifestyle No. (%)*	187 (33.6)
Unhealthy individual lifestyle No. (%)*	370 (66.4)
Healthy housing conditions No. (%)*	171 (30.6)
Unhealthy housing conditions No. (%)*	387 (69.4)
Median chronic air pollution exposures (IQR)	
NO_2_ (µg/m^3^)	29.8 (11.4)
NO_x_ (µg/m^3^)	47.7 (31.5)
PM_2.5_ (µg/m^3^)	25.4 (3.3)
PM_10_ (µg/m^3^)	38.3 (3.8)
PM_coarse_ (µg/m^3^)	13.2 (3.3)
PM_2.5 absorbance_ (10^-5^/m)	1.9 (0.7)
Individuals exposed to air pollution higher than the European Union threshold No. (%)	293 (55.9)

Chronic lung disease=any of the following conditions including asthma, chronic bronchitis, cough, cough with sputum or chronic obstructive pulmonary disease diagnosed by a physician.

Chronic air pollution exposure of 15 years before the respiratory assessments=average of annual mean exposures at the baseline and first follow-up examinations.

European Union threshold^37^=NO_2_: 40 µg/m3; PM_2.5_: 25 µg/m3; PM_10_: 40 µg/m3.

Missing data were excluded (3 individuals for passive smoking, 36 individuals for air pollution exposure, 3 individuals for lifestyle, 2 individuals for housing conditions and 1 individual for education level).

* Defined in the methods.

NO_2_, nitrogen dioxide; NO_x_, nitrogen oxides; PM_2.5_, particulate matter with median aerodynamic diameters <2.5; PM2.5/10/coarse, particulate matter with median aerodynamic diameters of ≤2.5/≤10/ 2.5–10 μm; SALIA, Study on the influence of Air pollution on Lung function, Inflammation and Aging.

**Figure 1:**
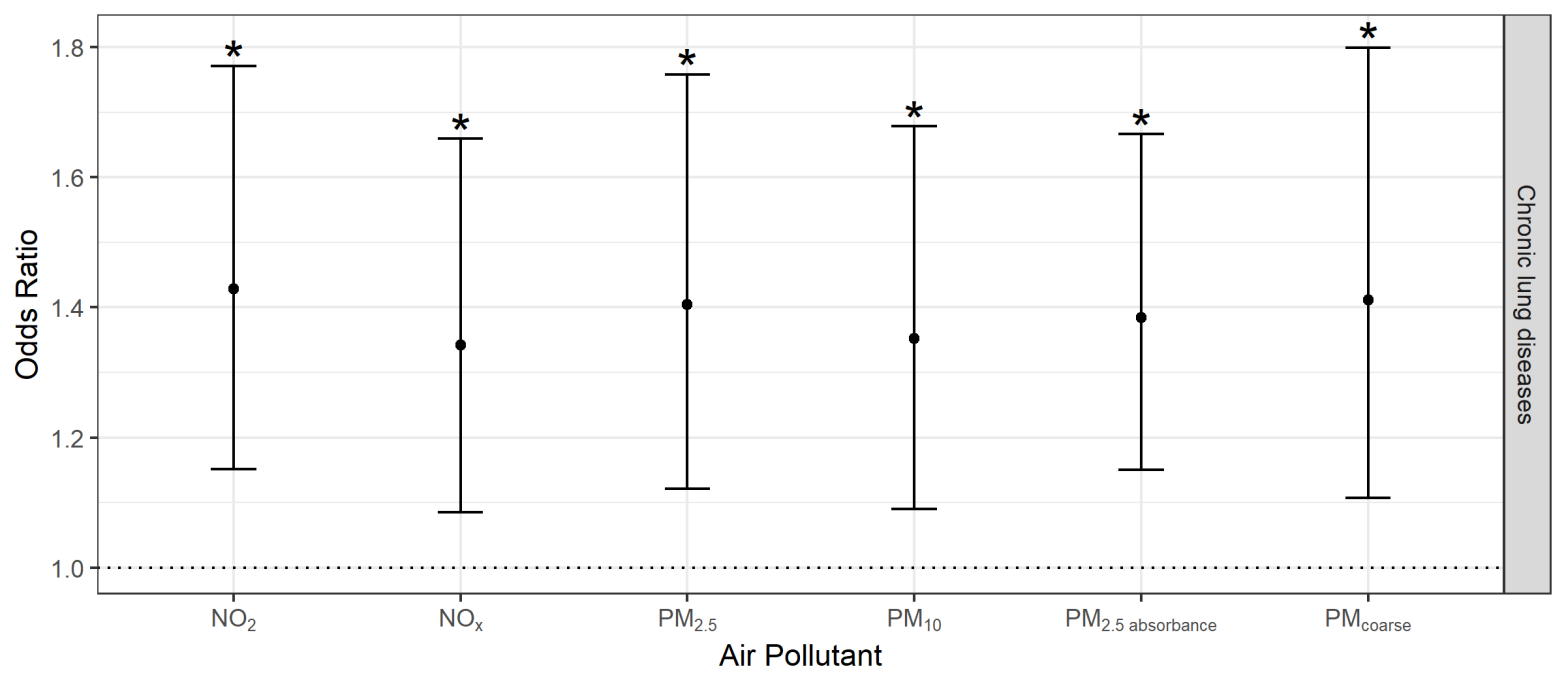
Environmental main effects on chronic lung disease. ORs and corresponding 95% CIs per 1 IQR increase in air pollution exposure adjusted for age, body mass index, educational level (low as the reference, medium, high), smoking status (never as the reference, ever), passive smoking (never as the reference, ever), indoor air pollution (dampness, mould or cooking with gas at home) and residential moving in the observation time. N=737 observations with complete data were used. Asterisks (*) indicate statistical significance (p<0.05). NO_2_, nitrogen dioxide; NO_X_, nitrogen oxides; PM_2.5_, particulate matter with median aerodynamic diameters <2.5.

No statistically significant association was observed between the GRS and chronic lung disease in the adjusted models (OR=1.11, 95% CI 0.851 to 1.461, p=0.428). However, higher exposures to each air pollutant were associated with increased adjusted odds of chronic lung disease by 34% (NO_x_: IQR=31.5 µg/m³, OR=1.342, 95% CI 1.086 to 1.659, p=0.007) to 43% per one IQR increase (NO_2_: IQR=11.4 µg/m³, OR=1.428, 95% CI 1.152 to 1.771, p=0.001) (figure 1, online supplemental table S2).

**Figure 2:**
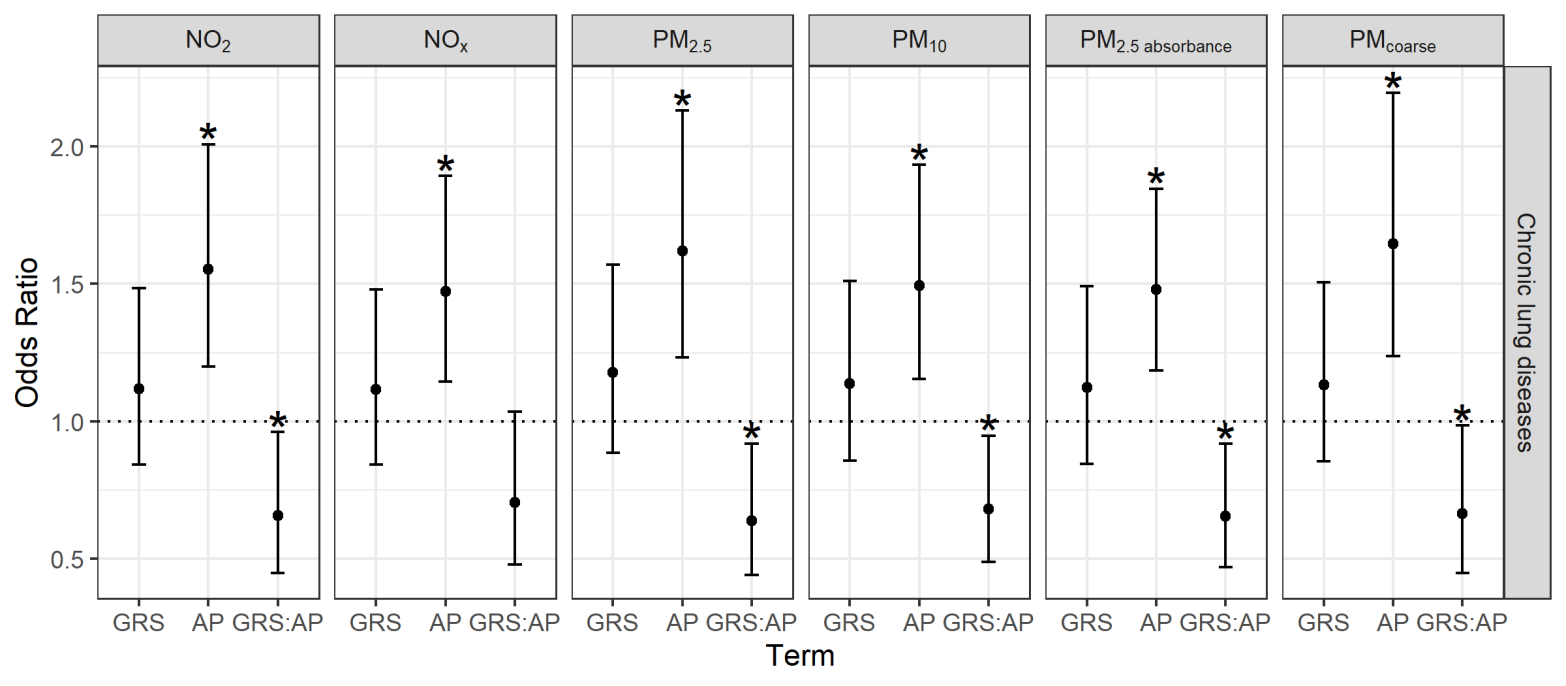
Gene–environment interaction effects on chronic lung disease. ORs and corresponding 95% CIs per 1 IQR increase in GRS or air pollution exposure adjusted for age, body mass index, educational level (low as the reference, medium, high), smoking status (never as the reference, ever), passive smoking (never as the reference, ever), indoor air pollution (dampness, mould or cooking with gas at home) and residential moving in the observation time. N=560 observations for GRS construction, N=520 observations with complete data for GxE interaction testing. Asterisks (*) indicate statistical significance (p<0.05). AP, air pollutant; GRS, genetic risk score; GRS:AP, interaction between GRS and AP; GxE, gene–environment interactions; NO_2_, nitrogen dioxide; NO_X_, nitrogen oxides; PM_2.5_, particulate matter with median aerodynamic diameters <2.5.

In the adjusted GxE models, air pollution effects remained stable and GxE effects were identified for all air pollutants except for NO_x_ (figure 2, online supplemental table S3). Similar results were observed in the crude GxE models (online supplemental table S4) and using a standard internal weighted GRS approach (online supplemental table S5).

**Figure 3:**
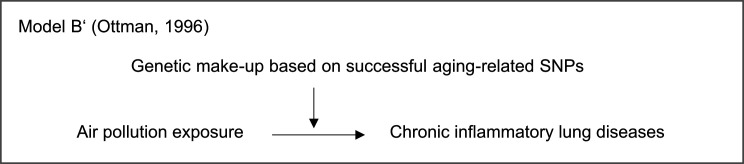
Modelled interaction between air pollution exposure and the individual genetic make-up on chronic lung disease applied by Ottman.^36^ SNPs, single-nucleotide polymorphisms.

The four risk subgroups showed that the relative risk of chronic lung disease in high air pollution exposed individuals (median split) presenting a high-risk GRS was lower than the multiplicative effect of less exposed individuals presenting a high-risk GRS and high exposed individuals presenting a low-risk GRS, which defined antagonistic GxE effects^36^ (online supplemental table S6). This meant that the GRS reduced the risk impact of air pollution exposure. Combining all results, the GRS attenuated the impact of the air pollution exposure, but had no main effect independent of the air pollutants (Ottman’s model B’, figure 3). In women exposed to air pollution exceeding the EU thresholds, the effects were robust (online supplemental table S7).

**Figure 4:**
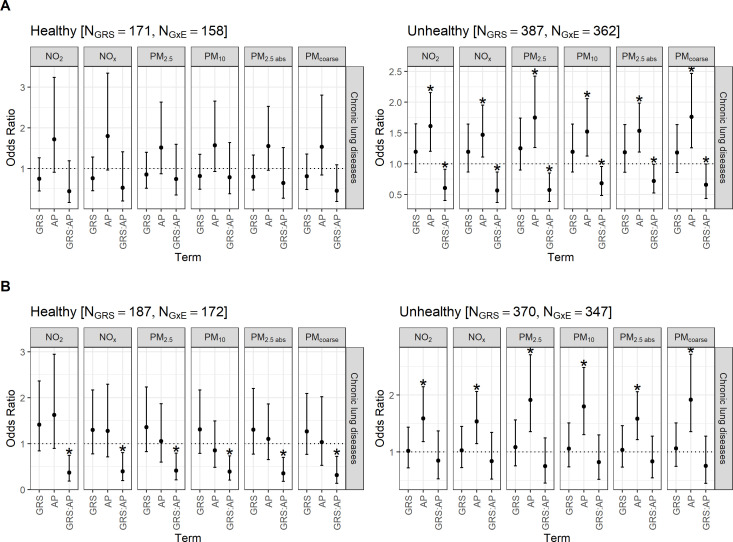
Gene–environment interaction effects on chronic lung disease in subgroups regarding healthy versus unhealthy (**A**) housing conditions and (**B**) individual lifestyle. ORs and corresponding 95% CIs per 1 IQR increase in GRS or air pollution exposure adjusted for age, body mass index, educational level (low as the reference, medium, high), smoking status (never as the reference, ever), passive smoking (never as the reference, ever), indoor air pollution (dampness, mould or cooking with gas at home) and residential moving in the observation time. Asterisks (*) indicate statistical significance (p<0.05). AP, air pollutant; GRS, genetic risk score; GRS:AP, interaction between GRS and AP; GxE, gene–environment interactions; N_GRS_, observations for GRS construction; N_GxE_, observations with complete data for GxE interaction testing; NO_2_, nitrogen dioxide; NO_X_, nitrogen oxides; PM_2.5_, particulate matter with median aerodynamic diameters <2.5.

In the subgroup analyses (figure 4, online supplemental tables S8–S13), we found no associations for women living in healthy housing, but for women living in unhealthy housing conditions, the associations of the main model remained stable. In women with an unhealthy lifestyle, the odds of chronic lung disease were increased by up to 92% per 1 IQR increase in air pollution, while the GRS did not attenuate the air pollution impact. However, in women with a healthy lifestyle, the air pollutants did not increase the odds of chronic lung disease, while the genetic make-up attenuated the impact of air pollution.

## Discussion

In this study, we have explored the interaction between genetic predisposition to successful ageing and air pollution exposure on chronic lung disease in a cohort of healthy aged German women. While we observed no genetic main effect, a higher exposure to air pollutants increased the odds of chronic lung disease in older ages. Furthermore, antagonistic GxE effects that attenuate the environmental main impact were identified. The genetic predisposition of the total sample, of women exposed to air pollution exceeding the EU thresholds, and of women living in unhealthy housing conditions reduces the impact of air pollution exposure, but had no effect independent of air pollution. However, women with an unhealthy lifestyle had higher air pollution impacts without the protective influence of their individual genetic predisposition. In contrast, the respiratory health of women with a healthy lifestyle was not affected by air pollutants, while the genetic predisposition decreased the risk impact of air pollution exposure.

### Interpretation of findings

The inflammatory and oxidative stress pathways have been proposed to explain associations between air pollution and respiratory health, whereby there might be an interaction with the individual genetic susceptibility^⁠^.^1 2^ Thus, in the context of GxE, lung diseases might be influenced by inflammation caused by air pollution which could be regulated by genetic make-up. Alternatively, air pollution exposure may affect the genetic susceptibility to respiratory health via inflammation.

Previous GxE studies on respiratory health focused on genetic variants related to respiratory health outcomes.^14 15^ Moreover, most of the epidemiological studies investigated adults, while only some included healthy elderlies. This study contributes new evidence of lung ageing in elderly women by examining successful ageing genetic variants. Candidate successful ageing genes have already been identified in GWAS and candidate gene studies, particularly the apolipoprotein E (APOE) and forkhead box O3 (FOXO3) loci^11^,^13^ (both covered in our study). However, most studies using ageing genetics focused on the genetic impact on age in centenarians who can avoid or live with age-related diseases^⁠^.^6 7 13^ A recent review suggested the use of disease-related SNPs in ageing studies due to the genetic overlap^⁠^.^6^ To the best of our knowledge, only one existing study examined the overlap the other way around by investigating genetic predisposition to successful ageing on respiratory diseases, where a higher GRS for lifespan was associated with fewer respiratory diseases.^13^ Furthermore, existing studies on ageing highlighted the interaction of genetics with the environment. Our study adds to the evidence by investigating associations between ageing genetic variants and air pollution exposures as existing studies did not include the physical, but only the social environment^⁠^.^5 6^

In our study, we found robust antagonistic GxE effects as the genetic predisposition based on successful ageing SNPs attenuated the impact of air pollution exposure, but had no impact without the air pollution exposure. This supports that lung diseases might be influenced by inflammation related to air pollution which is regulated by genetic make-up. Thus, it could not be supported that the environment of centenarians neutralises the risk effect of genetics^⁠^.^3 10^ In inflammatory-related diseases and the ageing process, the genetic role was described as less important than the environmental factors^⁠^.^5^

Underlying mechanisms of GxE might differ between subgroups.^36^ Many determinants for ageing and its genetics have already been identified^⁠^.^3 4 6 10^ Lifestyle is a component that interacts with the genetic make-up, so some individuals might have the capacity to reduce oxidative stress and inhibit the development of related chronic diseases^⁠^.^3 5 39^ One existing study found positive correlations between longevity and smoking cessation, while there are negative correlations with obesity, which they hypothesised to affect longevity through coronary artery disease.^29^

To our knowledge, no comparable study has included the lifestyle component as an additional dimension in a GxE analysis on respiratory health. Our study suggested that women with an unhealthy lifestyle were more affected by air pollution impacts, while there was no interaction with their genetic make-up. While women with a healthy lifestyle were not affected by risk-increasing air pollutants, their genetic predisposition attenuated the impact of the air pollution exposure. The finding may be explained by a potential influence of a healthy lifestyle, characterised by normal BMI, exercise and absence of smoking, on oxidative stress and inflammatory pathways. A subsequent question arising from this is whether individuals with a healthy lifestyle, such as maintaining a normal BMI, derive greater benefit from the genetic predisposition compared with those with elevated BMI, or alternatively, whether the genetic predisposition facilitates the maintenance of a normal BMI.

This study highlights the importance of a healthy environment, including reduction of air pollution and having a healthy individual lifestyle in the ageing population. These findings identified modifiable targets for public health strategies. Despite the non-significant findings of the GRS on chronic lung disease, GRSs generally have the potential for individual risk prediction and personalised prevention or therapy approaches. In combination with environmental factors, individuals may become more sensitive to disease risk, which could lead to behavioural or lifestyle changes to achieve successful ageing. Public health strategies could be targeted more precisely to individuals based on their GRS and evidence on GxE, which could contribute to a more efficient healthcare system. Further studies should take a broader perspective on ageing and chronic diseases in the elderly. Combining the evidence of both fields, including genetics, environment, lifestyle or additional components, could help to identify interaction effects to better understand the ageing process and chronic diseases.

### Strengths and limitations

This study has several strengths, including a well-characterised cohort. Using the SALIA cohort offers the chance to answer the research question by investigating an elderly group with more than 15 years of collected history of air pollution exposure. About 30% of the women already developed any chronic lung disease, and the successful ageing process likely already has taken effect. The air pollution exposure was assigned within the ESCAPE study, resulting in comparable, high-quality measured and modelled exposure data. The ageing-related SNPs fitted the study sample regarding ethnicity and age.^24 40^ Furthermore, SNPs that have a similar pathway might have strengthened the GRS.^41^ The GRS was based on recent candidate studies and GWAS on different ageing phenotypes. The applied construction method has the advantage that the sample did not have to be split, which would reduce statistical power. Applying a standard internal weighted GRS approach^33^ confirmed the robustness of our results, whereby not for all models could reasonable GRS be fitted and statistical power was lower due to the random data split. Sex-specific differences^27^ could be neglected, as this study only focused on women. In the statistical analysis, we included several potential confounders selected a priori to decrease the risk of bias.

However, some limitations have to be considered while interpreting the findings. Using cohort data included the risk of selection bias due to loss to follow-up. In our study, we did not exclude women with pre-existing diseases, did not investigate potential modifying effects of chronic diseases, and we did not analyse respiratory health longitudinally using more than one time point. Chronic lung disease was defined using self-responses of doctor diagnoses that could tend to underestimate the results. There could be other relevant environmental factors besides air pollutants, further determinants to define chronic lung disease or to characterise a healthy lifestyle.^39^ Furthermore, there might be an underestimation of effects if statistical power was not high enough due to the small sample size.

## Conclusions

This GxE interaction analysis in elderly women suggests that having a genetic predisposition based on successful ageing genetic variants reduces the negative impact of air pollution on chronic lung disease in older age, while a healthy lifestyle further strengthens this association.

## Supplementary material

10.1136/bmjresp-2025-003226 Supplementary file 1

## Data Availability

Data are available on reasonable request.
