# Supplementary material for: Interaction between genetic predisposition to successful ageing and chronic air pollution on lung disease in elderly women: results of the German SALIA cohort
Source: BMJ Open Respir Res. 2025 Nov 4;12(1):e003226. doi: 10.1136/bmjresp-2025-003226 (PMC12587969; doi:10.1136/bmjresp-2025-003226)
Supplement: Supplementary file 1 [file bmjresp-12-1-s001.docx]

**Supplementary Material**

**Interaction between genetic predisposition to successful ageing and chronic air pollution on lung disease in elderly women: results of the German SALIA cohort**

**Table of Contents**

| Table S1 | Information on 89 ageing-related single nucleotide polymorphisms from different candidate gene and genome-wide association studies. A total of 77 single nucleotide polymorphisms were included in the genetic risk score (marked bold). |
| --- | --- |
| Table S2 | Adjusted environmental main effects on chronic lung diseases. |
| Table S3 | Adjusted gene-environment interaction effects on chronic lung diseases. |
| Table S4 | Crude gene-environment interaction effects on chronic lung diseases. |
| Table S5 | Adjusted gene-environment interaction effects on chronic lung diseases using a standard internal weighted genetic risk score approach^26^. |
| Table S6 | Gene-environment interaction analysis using relative risks^27^ for chronic lung diseases in subgroups with genetic high-risk vs. low-risk air pollution exposed vs. unexposed (median). |
| Table S7 | Gene-environment interaction analysis using relative risks^27^ for chronic lung diseases in subgroups with genetic high-risk vs. low-risk air pollution exposed vs. unexposed (EU threshold). |
| Table S8 | Description of the study subgroups according to healthy or unhealthy individual lifestyles. |
| Table S9 | Adjusted gene-environment interaction effects on chronic lung diseases in elderly women with a healthy individual lifestyle. |
| Table S10 | Adjusted gene-environment interaction effects on chronic lung diseases in elderly women with an unhealthy individual lifestyle. |
| Table S11 | Description of the study subgroups according to healthy or unhealthy housing conditions. |
| Table S12 | Adjusted gene-environment interaction effects on chronic lung diseases in elderly women living in healthy housing conditions. |
| Table S13 | Adjusted gene-environment interaction effects on chronic lung diseases in elderly women living in unhealthy housing conditions. |

**Table S1: Information on 89 ageing-related single nucleotide polymorphisms from different candidate gene and genome-wide association studies. A total of 77 single nucleotide polymorphisms were included in the genetic risk score (marked bold).**

| **Chromosome** | **rsID** | **Candidate gene or genome-wide association study** | **Source(s)/ references** |
| --- | --- | --- | --- |
| 1 | **rs4611001** | RGS7 | ^1^ ^2^ |
| 1 | **rs4443878** | RGS7 | ^3^ ^2^ |
| 1 | **rs602633** | CELSR2 and PSRC1 | ^4^ ^5^ ^6^ |
| 1 | **rs11585386** | Aging | GCST001599 ^7^ |
| 1 | **rs11162963** | Aging (time to event) | GCST001166 ^8^ |
| 1 | **rs2367725** | Aging (time to event) | GCST001166 ^8^ |
| 1 | **rs12739243** | Frailty index | GCST90020053 ^9^ |
| 1 | rs3006879 | GWAS of female lifespan data (aging) | ^10^ |
| 2 | **rs12474609** | Aging | GCST000378 ^11^ |
| 2 | **rs10202497** | Aging (time to event) | GCST001166 ^8^ |
| 2 | **rs4952693** | Frailty index | GCST90020053 ^9^ |
| 3 | **rs9825185** | C3orf21 | ^3^ ^2^ |
| 3 | **rs9841144** | CADM2 | ^1^ ^2^ |
| 3 | **rs322458** | Aging (facial) | GCST001767 ^12^ |
| 3 | **rs1425609** | Aging (time to death) | GCST001167 ^8^ |
| 3 | **rs16852912** | Aging (time to event) | GCST001166 ^8^ |
| 3 | **rs3772255** | Aging traits | GCST000096 ^13^ |
| 3 | **rs2071207** | Frailty index | GCST90020053 ^9^ |
| 3 | **rs583514** | Frailty index | GCST90020053 ^9^ |
| 4 | **rs82334** | Frailty index | GCST90020053 ^9^ |
| 5 | **rs2149954** | 5q33.3/EBF1; LINC02227 | ^14^ ^2^ ^5^ ^6^ |
| 5 | **rs10491334** | CAMK4 | ^15^ ^2^ |
| 5 | **rs2706372** | RAD50 and IL13 | ^16^ ^5^ ^6^ |
| 5 | **rs294588** | Aging | GCST001599 ^7^ |
| 5 | **rs3112530** | Aging (time to event) | GCST001166 ^8^ |
| 5 | **rs1363103** | Frailty index | GCST90020053 ^9^ |
| 6 | **rs3800231** | FOXO3 | ^16^ ^2^ |
| 6 | **rs2802292** | FOXO3 | ^17^ ^2^ ^1^ ^5^ ^6^ ^18^ |
| 6 | **rs10457180** | FOXO3 | ^1^ ^2^ |
| 6 | **rs954551** | GRIK2 | ^3^ ^2^ |
| 6 | **rs1416280** | GRIK2 | ^1^ ^2^ |
| 6 | **rs55730499** | LPA/ APOE | ^2^ ^4^ ^5^ ^6^ |
| 6 | **rs28383322** | HLA-DQA1 and HLA-DRB1 | ^4^ ^5^ ^6^ |
| 6 | **rs34831921** | HLA-DQA1 and HLA-DRB1 | ^2^ ^5^ ^6^ |
| 6 | **rs2253310** | FOXO3 | ^18^ |
| 6 | **rs4946936** | FOXO3 | ^18^ |
| 6 | **rs1572438** | Aging | GCST001599 ^7^ |
| 6 | **rs6925255** | Aging | GCST001599 ^7^ |
| 6 | **rs1247318** | Aging | GCST001599 ^7^ |
| 6 | **rs16892673** | Aging | GCST001599 ^7^ |
| 6 | **rs9275160** | Frailty index | GCST90020053 ^9^ |
| 7 | **rs2069837** | IL6 | ^19^ ^2^ |
| 7 | **rs3764814** | USP42 | ^20^ ^5^ ^6^ |
| 7 | **rs9918668** | Aging | GCST001599 ^7^ |
| 7 | **rs2396766** | Frailty index | GCST90020053 ^9^ |
| 7 | rs2229188 | GWAS of female lifespan data | ^10^ |
| 8 | **rs7844965** | EPHX2 | ^4^ ^5^ ^6^ |
| 8 | **rs56299474** | Frailty index | GCST90020053 ^9^ |
| 8 | rs11574358 | GWAS of female lifespan data | ^10^ |
| 9 | **rs1333049** | CDKN2A/B | ^21^ ^2^ |
| 9 | **rs4977756** | CDKN2A/B | ^22^ ^2^ |
| 9 | **rs514659** | ABO | ^22^ ^2^ |
| 9 | **rs1556516** | CDKN2A and CDKN2B | ^4^ ^5^ ^6^ |
| 10 | **rs9664222** | MINPP1 | ^3^ ^2^ |
| 10 | rs4746720 | SIRT1_720 | ^23^ |
| 10 | **rs4146140** | Frailty index | GCST90020053 ^9^ |
| 10 | **rs7894051** | GWAS of female lifespan data, selected to investigate association with CVD | ^10^ |
| 11 | rs139137459 | USP2-AS1 | ^4^ ^5^ ^6^ |
| 11 | **rs4936894** | Aging (time to death) | GCST001167 ^8^ |
| 11 | **rs10891490** | Frailty index | GCST90020053 ^9^ |
| 11 | rs4639950 | GWAS of female lifespan data | ^10^ |
| 11 | rs217237 | GWAS of female lifespan data | ^10^ |
| 12 | **rs3184504** | SH2B3 | ^22^ ^2^ |
| 12 | **rs7976168** | TMTC2 | ^20^ ^5^ ^6^ |
| 12 | **rs7137828** | SH2B3/ATXN2 | ^4^ ^5^ ^6^ |
| 12 | **rs643473** | Aging | GCST001599 ^7^ |
| 12 | **rs766903** | Aging (time to death) | GCST001167 ^8^ |
| 12 | **rs4764043** | Aging (time to event) | GCST001166 ^8^ |
| 12 | **rs1463605** | Aging traits | GCST000096 ^13^ |
| 12 | rs2292664 | GWAS of female lifespan data | ^10^ |
| 13 | rs2440012 | ANKRD20A9P | ^19^ ^2^ |
| 13 | **rs8001976** | Aging (time to event) | GCST001166 ^8^ |
| 14 | **rs61978928** | PROX2 | ^4^ ^5^ ^6^ |
| 14 | rs4904670 | GWAS of female lifespan data, selected to investigate association with CVD | ^10^ |
| 15 | **rs1317286** | CHRNA3 and CHRNA5 | ^4^ ^5^ ^6^ |
| 15 | **rs8042849** | CHRNA3 and CHRNA5, APOE | ^2^ ^5^ ^6^ |
| 15 | **rs17514846** | FURIN | ^4^ ^5^ ^6^ |
| 15 | **rs7176093** | Aging traits | GCST000096 ^13^ |
| 15 | **rs3959554** | Frailty index | GCST90020053 ^9^ |
| 15 | **rs17612102** | Frailty index | GCST90020053 ^9^ |
| 17 | rs12949468 | GWAS of female lifespan data | ^10^ |
| 18 | **rs28926173** | MC2R | ^4^ ^5^ ^6^ |
| 18 | **rs8089807** | Frailty index | GCST90020053 ^9^ |
| 19 | **rs6857** | APOE | ^20^ ^5^ ^6^ |
| 19 | **rs4420638** | APOE | ^14^ ^24^ ^5^ ^6^ |
| 19 | **rs429358** | APOE | ^2^ ^5^ ^6^ |
| 19 | **rs2075650** | APOE | ^16^ ^5^ ^6^ |
| 19 | **rs10412199** | Aging (time to event) | GCST001166 ^8^ |
| 19 | rs5491 | GWAS of female lifespan data | ^10^ |
| *rsID* reference single nucleotide polymorphisms cluster ID  *GCST…=* NHGRI-EBI GWAS Catalog ^25^ accession ID | | | |

**Table S2: Adjusted environmental main effects on chronic lung diseases.**

| **Air Pollutant** | **Odds Ratio** | **Odds Ratio 95% CI** | **beta** | **beta 95% CI** | **P** |
| --- | --- | --- | --- | --- | --- |
| NO_2_ | 1.428 | 1.152;1.771 | 0.356 | 0.141;0.572 | 0.001* |
| NO_x_ | 1.342 | 1.086;1.659 | 0.294 | 0.082;0.506 | 0.007* |
| PM_2.5_ | 1.404 | 1.122;1.758 | 0.340 | 0.115;0.564 | 0.003* |
| PM_10_ | 1.353 | 1.090;1.678 | 0.302 | 0.087;0.518 | 0.006* |
| PM_2.5 absorbance_ | 1.385 | 1.151;1.666 | 0.326 | 0.140;0.511 | 0.001* |
| PM_coarse_ | 1.412 | 1.108;1.799 | 0.345 | 0.102;0.587 | 0.005* |
| Effect estimates per one interquartile range increase in air pollution exposure adjusted for age, body mass index, educational level (low as the reference, medium, high), smoking status (never as the reference, ever), passive smoking (never as the reference, ever), indoor air pollution (dampness, mould or cooking with gas at home), and residential moving in the observation time. N=737 observations with complete data were utilized.  CI=confidence intervals, NO_2_=nitrogen dioxide, NO_x_=nitrogen oxides, PM_2.5/10/coarse_=particulate matter with median aerodynamic diameters of ≤2.5/≤10/ 2.5–10 μm, PM_2.5 absorbance=_ the reflectance of PM_2.5_ filters, *=P<0.05 | | | | | |

**Table S3: Adjusted gene-environment interaction effects on chronic lung diseases.**

| **Air Pollutant** | **Term** | **Odds Ratio** | **Odds Ratio 95% CI** | **beta** | **beta 95% CI** | **P** |
| --- | --- | --- | --- | --- | --- | --- |
| NO_2_ | GRS | 1.119 | 0.843;1.485 | 0.112 | -0.171;0.395 | 0.438 |
| NO_2_ | AP | 1.552 | 1.200;2.008 | 0.440 | 0.182;0.697 | 0.001* |
| NO_2_ | GRS:AP | 0.657 | 0.449;0.961 | -0.420 | -0.801;-0.040 | 0.030* |
| NO_x_ | GRS | 1.116 | 0.842;1.479 | 0.110 | -0.172;0.391 | 0.446 |
| NO_x_ | AP | 1.472 | 1.145;1.892 | 0.387 | 0.136;0.638 | 0.003* |
| NO_x_ | GRS:AP | 0.704 | 0.479;1.036 | -0.351 | -0.737;0.035 | 0.075 |
| PM_2.5_ | GRS | 1.179 | 0.885;1.571 | 0.164 | -0.123;0.452 | 0.261 |
| PM_2.5_ | AP | 1.620 | 1.233;2.129 | 0.483 | 0.209;0.756 | 0.001* |
| PM_2.5_ | GRS:AP | 0.638 | 0.442;0.919 | -0.450 | -0.816;-0.084 | 0.016* |
| PM_10_ | GRS | 1.138 | 0.857;1.511 | 0.129 | -0.155;0.413 | 0.373 |
| PM_10_ | AP | 1.493 | 1.154;1.932 | 0.401 | 0.143;0.659 | 0.002* |
| PM_10_ | GRS:AP | 0.681 | 0.490;0.948 | -0.384 | -0.714;-0.054 | 0.023* |
| PM_2.5 absorbance_ | GRS | 1.124 | 0.846;1.492 | 0.117 | -0.167;0.400 | 0.421 |
| PM_2.5 absorbance_ | AP | 1.480 | 1.186;1.846 | 0.392 | 0.170;0.613 | 0.001* |
| PM_2.5 absorbance_ | GRS:AP | 0.656 | 0.469;0.918 | -0.421 | -0.757;-0.086 | 0.014* |
| PM_coarse_ | GRS | 1.134 | 0.854;1.505 | 0.125 | -0.158;0.409 | 0.385 |
| PM_coarse_ | AP | 1.647 | 1.237;2.194 | 0.499 | 0.213;0.786 | 0.001* |
| PM_coarse_ | GRS:AP | 0.664 | 0.447;0.985 | -0.410 | -0.804;-0.015 | 0.042* |
| Effect estimates per one interquartile range increase in GRS or air pollution exposure adjusted for age, body mass index, educational level (low as the reference, medium, high), smoking status (never as the reference, ever), passive smoking (never as the reference, ever), indoor air pollution (dampness, mould or cooking with gas at home), and residential moving in the observation time. N=560 observations for GRS construction, N=520 observations with complete data for GxE interaction testing.  AP=Air pollutant, CI=confidence intervals, GRS=Genetic risk score, GRS:AP=Interaction between GRS and AP, NO_2_=nitrogen dioxide, NO_x_=nitrogen oxides, PM_2.5/10/coarse_=particulate matter with median aerodynamic diameters of ≤2.5/≤10/ 2.5–10 μm, PM_2.5 absorbance=_ the reflectance of PM_2.5_ filters,*=P<0.05 | | | | | | |

**Table S4: Crude gene-environment interaction effects on chronic lung diseases.**

| **Air Pollutant** | **Term** | **Odds Ratio** | **Odds Ratio 95% CI** | **beta** | **beta 95% CI** | **P** |
| --- | --- | --- | --- | --- | --- | --- |
| NO_2_ | GRS | 1.107 | 0.837;1.463 | 0.101 | -0.178;0.381 | 0.477 |
| NO_2_ | AP | 1.477 | 1.162;1.879 | 0.390 | 0.150;0.631 | 0.001* |
| NO_2_ | GRS:AP | 0.661 | 0.454;0.961 | -0.415 | -0.790;-0.040 | 0.030* |
| NO_x_ | GRS | 1.102 | 0.835;1.455 | 0.097 | -0.181;0.375 | 0.493 |
| NO_x_ | AP | 1.437 | 1.133;1.823 | 0.362 | 0.125;0.600 | 0.003* |
| NO_x_ | GRS:AP | 0.707 | 0.483;1.035 | -0.347 | -0.728;0.034 | 0.075 |
| PM_2.5_ | GRS | 1.165 | 0.878;1.547 | 0.153 | -0.130;0.436 | 0.290 |
| PM_2.5_ | AP | 1.472 | 1.138;1.904 | 0.386 | 0.129;0.644 | 0.003* |
| PM_2.5_ | GRS:AP | 0.624 | 0.434;0.897 | -0.471 | -0.834;-0.109 | 0.011* |
| PM_10_ | GRS | 1.125 | 0.851;1.489 | 0.118 | -0.162;0.398 | 0.408 |
| PM_10_ | AP | 1.337 | 1.059;1.688 | 0.291 | 0.058;0.523 | 0.014* |
| PM_10_ | GRS:AP | 0.661 | 0.475;0.920 | -0.414 | -0.745;-0.083 | 0.014* |
| PM_2.5 absorbance_ | GRS | 1.111 | 0.840;1.469 | 0.105 | -0.175;0.385 | 0.462 |
| PM_2.5 absorbance_ | AP | 1.421 | 1.149;1.758 | 0.352 | 0.139;0.564 | 0.001* |
| PM_2.5 absorbance_ | GRS:AP | 0.663 | 0.476;0.923 | -0.411 | -0.741;-0.080 | 0.015* |
| PM_coarse_ | GRS | 1.118 | 0.846;1.478 | 0.111 | -0.167;0.390 | 0.434 |
| PM_coarse_ | AP | 1.507 | 1.152;1.972 | 0.410 | 0.141;0.679 | 0.003* |
| PM_coarse_ | GRS:AP | 0.658 | 0.444;0.973 | -0.419 | -0.811;-0.027 | 0.036* |
| Effect estimates per one interquartile range increase in GRS or air pollution exposure without adjustment for potential confounders. N=560 observations for GRS construction, N=524 observations with genetic and air pollution data for GxE interaction testing.  AP=Air pollutant, CI=confidence intervals, GRS=Genetic risk score, GRS:AP=Interaction between GRS and AP, NO_2_=nitrogen dioxide, NO_x_=nitrogen oxides, PM_2.5/10/coarse_=particulate matter with median aerodynamic diameters of ≤2.5/≤10/ 2.5–10 μm, PM_2.5 absorbance=_ the reflectance of PM_2.5_ filters,*=P<0.05 | | | | | | |

**Table S5: Adjusted gene-environment interaction effects on chronic lung diseases using a standard internal weighted genetic risk score approach^26^.**

| **Air Pollutant** | **Term** | **Odds Ratio** | **Odds Ratio 95% CI** | **beta** | **beta 95% CI** | **P** |
| --- | --- | --- | --- | --- | --- | --- |
| NO_2_ | GRS | 0.883 | 0.545;1.432 | -0.124 | -0.608;0.359 | 0.615 |
| NO_2_ | AP | 1.566 | 1.062;2.309 | 0.449 | 0.060;0.837 | 0.024* |
| NO_2_ | GRS:AP | 0.538 | 0.289;0.999 | -0.620 | -1.240;-0.001 | 0.050* |
| NO_x_ | AP | 1.395 | 0.955;2.037 | 0.333 | -0.046;0.711 | 0.085 |
| PM_2.5_ | AP | 1.452 | 1.000;2.108 | 0.373 | 0.000;0.746 | 0.050* |
| PM_10_ | AP | 1.507 | 1.029;2.208 | 0.410 | 0.028;0.792 | 0.035* |
| PM_2.5 absorbance_ | GRS | 0.891 | 0.558;1.422 | -0.116 | -0.583;0.352 | 0.628 |
| PM_2.5 absorbance_ | AP | 1.373 | 0.996;1.893 | 0.317 | -0.004;0.638 | 0.053 |
| PM_2.5 absorbance_ | GRS:AP | 0.800 | 0.449;1.425 | -0.223 | -0.800;0.354 | 0.449 |
| PM_coarse_ | AP | 2.005 | 1.339;3.003 | 0.696 | 0.292;1.100 | 0.001* |
| Effect estimates per one interquartile range increase in GRS or air pollution exposure adjusted for age, body mass index, educational level (low as the reference, medium, high), smoking status (never as the reference, ever), passive smoking (never as the reference, ever), indoor air pollution (dampness, mould or cooking with gas at home), and residential moving in the observation time.  For NO_x,_ PM_2.5_, PM_10_, and PM_coarse_ no reasonable GRS could be fitted due to the random (50:50) data split (which was repeated for each air pollutant) required for the standard internal weighted GRS approach.  AP=Air pollutant, CI=confidence intervals, GRS=Genetic risk score, GRS:AP=Interaction between GRS and AP, NO_2_=nitrogen dioxide, NO_x_=nitrogen oxides, PM_2.5/10/coarse_=particulate matter with median aerodynamic diameters of ≤2.5/≤10/ 2.5–10 μm, PM_2.5 absorbance=_ the reflectance of PM_2.5_ filters,*=P<0.05 | | | | | | |

**Table S6: Gene-environment interaction analysis using relative risks^27^ for chronic lung diseases in subgroups with genetic high-risk vs. low-risk air pollution high exposed vs. less exposed (median).**

| **Air Pollutant** | $\boldsymbol{R}\boldsymbol{R}_{\boldsymbol{11}}$ | $\boldsymbol{R}\boldsymbol{R}_{\boldsymbol{01}}\boldsymbol{+}\boldsymbol{R}\boldsymbol{R}_{\boldsymbol{10}}\boldsymbol{-}\boldsymbol{1}$ | $\boldsymbol{R}\boldsymbol{R}_{\boldsymbol{01}}\boldsymbol{\times}\boldsymbol{R}\boldsymbol{R}_{\boldsymbol{10}}$ | $\boldsymbol{R}\boldsymbol{R}_{\boldsymbol{01}}$ | $\boldsymbol{R}\boldsymbol{R}_{\boldsymbol{10}}$ | $\boldsymbol{R}\boldsymbol{R}_{\boldsymbol{00}}$ |
| --- | --- | --- | --- | --- | --- | --- |
| NO_2_ | 2.003 | 3.143 | 4.214 | 1.795 | 2.348 | 1.000 |
| NO_x_ | 2.264 | 3.360 | 4.619 | 1.814 | 2.546 | 1.000 |
| PM_2.5_ | 1.631 | 2.091 | 2.368 | 1.401 | 1.690 | 1.000 |
| PM_10_ | 1.325 | 1.456 | 1.508 | 1.231 | 1.226 | 1.000 |
| PM_coarse_ | 1.671 | 2.634 | 3.283 | 1.683 | 1.951 | 1.000 |
| PM_2.5 absorbance_ | 1.761 | 2.275 | 2.644 | 1.446 | 1.829 | 1.000 |
| Both genetic risk scores (GRS) and air pollutants (AP) have been divided into low and high risk using their respective medians. N=524 observations with genetic and air pollution data were utilized.  NO_2_=nitrogen dioxide, NO_x_=nitrogen oxides, PM_2.5/10/coarse_=particulate matter with median aerodynamic diameters of ≤2.5/≤10/ 2.5–10 μm, PM_2.5 absorbance=_ the reflectance of PM_2.5_ filters,$RR_{11}$: Relative risks for high AP exposure and high GRS, $RR_{01}$: Relative risks for low AP exposure and high GRS, $RR_{10}$: Relative risks for high AP exposure and low GRS, $RR_{00}$: Relative risks for low AP exposure and low GRS, $RR_{01}+RR_{10}-1$: Additive model, $RR_{01}\times RR_{10}$: Multiplicative model, *=P<0.05 | | | | | | |

**Table S7: Gene-environment interaction analysis using relative risks^27^ for chronic lung diseases in subgroups with genetic high-risk vs. low-risk air pollution high exposed vs. less exposed (EU threshold).**

| **Air Pollutant** | $\boldsymbol{R}\boldsymbol{R}_{\boldsymbol{11}}$ | $\boldsymbol{R}\boldsymbol{R}_{\boldsymbol{01}}\boldsymbol{+}\boldsymbol{R}\boldsymbol{R}_{\boldsymbol{10}}\boldsymbol{-}\boldsymbol{1}$ | $\boldsymbol{R}\boldsymbol{R}_{\boldsymbol{01}}\boldsymbol{\times}\boldsymbol{R}\boldsymbol{R}_{\boldsymbol{10}}$ | $\boldsymbol{R}\boldsymbol{R}_{\boldsymbol{01}}$ | $\boldsymbol{R}\boldsymbol{R}_{\boldsymbol{10}}$ | $\boldsymbol{R}\boldsymbol{R}_{\boldsymbol{00}}$ |
| --- | --- | --- | --- | --- | --- | --- |
| NO_2_ | 1.555 | 2.205 | 2.468 | 1.286 | 1.919 | 1.000 |
| PM_2.5_ | 1.764 | 2.453 | 2.962 | 1.591 | 1.862 | 1.000 |
| PM_10_ | 1.425 | 2.053 | 2.287 | 1.319 | 1.734 | 1.000 |
| Genetic risk scores (GRS) have been divided into low and high using its median. Air pollutants (AP) have been divided into low and high exposures using the respective EU thresholds (NO_2_: 40 µg/m^3^; PM_2.5_: 25 µg/m^3^; PM_10_: 40 µg/m^3^ ^28^). N=524 observations with genetic and air pollution data were utilized.  NO_2_=nitrogen dioxide, PM_2.5/10_=particulate matter with median aerodynamic diameters of ≤2.5/≤10 μm,$RR_{11}$: Relative risks for high AP exposure and high GRS, $RR_{01}$: Relative risks for low AP exposure and high GRS, $RR_{10}$: Relative risks for high AP exposure and low GRS, $RR_{00}$: Relative risks for low AP exposure and low GRS, $RR_{01}+RR_{10}-1$: Additive model, $RR_{01}\times RR_{10}$: Multiplicative model, *=P<0.05 | | | | | | |

**Table S8: Description of the study subgroups according to healthy or unhealthy individual lifestyles.**

|  | **Healthy** | **Unhealthy** |
| --- | --- | --- |
| N | 187 | 370 |
| Chronic lung diseases No. (%) | 46 (24.6) | 108 (29.2) |
| Mean age [years] ± SD | 72.5 ± 2.9 | 74.0 ± 2.9 |
| Mean Body Mass Index [kg/m²] ± SD | 25.1 ± 2.4 | 28.4 ± 4.7 |
| Educational level of the participant or spouse No. (%): | | |
| Less than 10 years | 30 (16.0) | 66 (17.9) |
| 10 years | 93 (49.7) | 184 (49.9) |
| More than 10 years | 64 (34.2) | 119 (32.2) |
| Smoking No. (%): | | |
| ever smoker | 0 (0.0) | 104 (28.1) |
| passive smoking | 112 (60.2) | 227 (61.7) |
| Indoor air pollution No. (%) | 50 (26.7) | 88 (23.8) |
| Residential move No. (%) | 19 (10.2) | 51 (13.8) |
| Median chronic air pollution exposure [IQR]: | | |
| NO_2_ [µg/m^3^] | 27.3 [10.5] | 31.5 [11.2] |
| NO_x_ [µg/m^3^] | 42.3 [24.4] | 53.6 [32.4] |
| PM_2.5_ [µg/m^3^] | 24.8 [5.5] | 25.7 [3.1] |
| PM_10_ [µg/m^3^] | 37.2 [8.1] | 38.6 [3.7] |
| PM_coarse_ [µg/m^3^] | 12.6 [3.5] | 13.4 [3.1] |
| PM_2.5 absorbance_ [10^-5^/m] | 1.8 [0.7] | 2.0 [0.6] |
| Individuals exposed to air pollution higher than the European Union threshold No. (%) | 81 (46.8) | 211 (60.3) |
| Healthy individual lifestyle= BMI< 30kg/m², never smoking, >0 hours sport and walking/riding at least 15 minutes per week  Chronic lung diseases=any of the following conditions: asthma, chronic bronchitis, cough (with sputum), or chronic obstructive pulmonary disease ever diagnosed by a physician at the second follow-up examination  Indoor air pollution=dampness, mould or cooking with gas at home  Chronic air pollution exposure of 15 years before the respiratory assessments= average of annual mean exposures at the baseline and first follow-up examinations  European Union threshold=NO_2_: 40 µg/m^3^; PM_2.5_: 25 µg/m^3^; PM_10_: 40 µg/m^3^ ^28^  IQR=interquartile ranges, NO_2_=nitrogen dioxide, NO_x_=nitrogen oxides, PM_2.5/10/coarse_=particulate matter with median aerodynamic diameters of ≤2.5/≤10/ 2.5–10 μm, PM_2.5 absorbance=_ the reflectance of PM_2.5_ filters, SD=standard deviation | | |

**Table S9: Adjusted gene-environment interaction effects on chronic lung diseases in elderly women with a healthy individual lifestyle.**

| **Air Pollutant** | **Term** | **Odds Ratio** | **Odds Ratio 95% CI** | **beta** | **beta 95% CI** | **P** |
| --- | --- | --- | --- | --- | --- | --- |
| NO_2_ | GRS | 1.411 | 0.844;2.361 | 0.345 | -0.170;0.859 | 0.189 |
| NO_2_ | AP | 1.623 | 0.895;2.945 | 0.484 | -0.111;1.080 | 0.111 |
| NO_2_ | GRS:AP | 0.373 | 0.187;0.745 | -0.986 | -1.677;-0.295 | 0.005* |
| NO_x_ | GRS | 1.296 | 0.776;2.167 | 0.260 | -0.254;0.773 | 0.322 |
| NO_x_ | AP | 1.278 | 0.712;2.295 | 0.246 | -0.339;0.831 | 0.411 |
| NO_x_ | GRS:AP | 0.397 | 0.196;0.804 | -0.923 | -1.628;-0.218 | 0.010* |
| PM_2.5_ | GRS | 1.359 | 0.827;2.233 | 0.307 | -0.190;0.803 | 0.226 |
| PM_2.5_ | AP | 1.056 | 0.597;1.868 | 0.054 | -0.516;0.625 | 0.851 |
| PM_2.5_ | GRS:AP | 0.414 | 0.216;0.795 | -0.882 | -1.533;-0.230 | 0.008* |
| PM_10_ | GRS | 1.307 | 0.789;2.167 | 0.268 | -0.237;0.773 | 0.298 |
| PM_10_ | AP | 0.851 | 0.486;1.492 | -0.161 | -0.722;0.400 | 0.574 |
| PM_10_ | GRS:AP | 0.392 | 0.210;0.734 | -0.936 | -1.562;-0.310 | 0.003* |
| PM_2.5 absorbance_ | GRS | 1.304 | 0.773;2.199 | 0.265 | -0.257;0.788 | 0.320 |
| PM_2.5 absorbance_ | AP | 1.103 | 0.652;1.864 | 0.098 | -0.427;0.623 | 0.715 |
| PM_2.5 absorbance_ | GRS:AP | 0.356 | 0.180;0.704 | -1.033 | -1.715;-0.351 | 0.003* |
| PM_coarse_ | GRS | 1.265 | 0.765;2.091 | 0.235 | -0.267;0.738 | 0.359 |
| PM_coarse_ | AP | 1.032 | 0.528;2.018 | 0.032 | -0.639;0.702 | 0.926 |
| PM_coarse_ | GRS:AP | 0.315 | 0.137;0.726 | -1.155 | -1.990;-0.321 | 0.007* |
| Healthy individual lifestyle= BMI< 30kg/m², never smoking, >0 hours sport and walking/riding at least 15 minutes per week,  Effect estimates per one interquartile range increase in GRS or air pollution exposure adjusted for age, body mass index, educational level (low as the reference, medium, high), smoking status (never as the reference, ever), passive smoking (never as the reference, ever), indoor air pollution (dampness, mould or cooking with gas at home), and residential moving in the observation time. N=187 observations for GRS construction, N=172 observations with complete data for GxE interaction testing.  AP=Air pollutant, CI=confidence intervals, GRS=Genetic risk score, GRS:AP=Interaction between GRS and AP, NO_2_=nitrogen dioxide, NO_x_=nitrogen oxides, PM_2.5/10/coarse_=particulate matter with median aerodynamic diameters of ≤2.5/≤10/ 2.5–10 μm, PM_2.5 absorbance=_ the reflectance of PM_2.5_ filters,*=P<0.05 | | | | | | |

**Table S10:** **Adjusted gene-environment interaction effects on chronic lung diseases in elderly women with an unhealthy individual lifestyle.**

| **Air Pollutant** | **Term** | **Odds Ratio** | **Odds Ratio 95% CI** | **beta** | **beta 95% CI** | **P** |
| --- | --- | --- | --- | --- | --- | --- |
| NO_2_ | GRS | 1.017 | 0.720;1.435 | 0.017 | -0.328;0.361 | 0.925 |
| NO_2_ | AP | 1.588 | 1.179;2.140 | 0.463 | 0.165;0.761 | 0.002* |
| NO_2_ | GRS:AP | 0.850 | 0.528;1.371 | -0.162 | -0.639;0.315 | 0.506 |
| NO_x_ | GRS | 1.026 | 0.727;1.448 | 0.026 | -0.319;0.370 | 0.883 |
| NO_x_ | AP | 1.535 | 1.144;2.061 | 0.429 | 0.134;0.723 | 0.004* |
| NO_x_ | GRS:AP | 0.840 | 0.525;1.344 | -0.174 | -0.644;0.295 | 0.467 |
| PM_2.5_ | GRS | 1.086 | 0.756;1.562 | 0.083 | -0.280;0.446 | 0.655 |
| PM_2.5_ | AP | 1.913 | 1.354;2.702 | 0.648 | 0.303;0.994 | 0.000* |
| PM_2.5_ | GRS:AP | 0.752 | 0.454;1.245 | -0.285 | -0.789;0.219 | 0.268 |
| PM_10_ | GRS | 1.057 | 0.740;1.510 | 0.056 | -0.301;0.412 | 0.760 |
| PM_10_ | AP | 1.797 | 1.302;2.479 | 0.586 | 0.264;0.908 | 0.000* |
| PM_10_ | GRS:AP | 0.823 | 0.522;1.297 | -0.195 | -0.650;0.260 | 0.400 |
| PM_2.5 absorbance_ | GRS | 1.035 | 0.733;1.461 | 0.034 | -0.311;0.379 | 0.846 |
| PM_2.5 absorbance_ | AP | 1.582 | 1.218;2.055 | 0.459 | 0.197;0.720 | 0.001* |
| PM_2.5 absorbance_ | GRS:AP | 0.835 | 0.546;1.278 | -0.180 | -0.606;0.245 | 0.406 |
| PM_coarse_ | GRS | 1.062 | 0.748;1.509 | 0.061 | -0.290;0.411 | 0.735 |
| PM_coarse_ | AP | 1.917 | 1.356;2.710 | 0.651 | 0.304;0.997 | 0.000* |
| PM_coarse_ | GRS:AP | 0.758 | 0.450;1.278 | -0.277 | -0.799;0.246 | 0.299 |
| Unhealthy individual lifestyle= BMI≥ 30kg/m², ever smoking, no  sport, or walking/riding less than 15 minutes per week,  Effect estimates per one interquartile range increase in GRS or air pollution exposure adjusted for age, body mass index, educational level (low as the reference, medium, high), smoking status (never as the reference, ever), passive smoking (never as the reference, ever), indoor air pollution (dampness, mould or cooking with gas at home), and residential moving in the observation time. N=370 observations for GRS construction, N=347 observations with complete data for GxE interaction testing.  AP=Air pollutant, CI=confidence intervals, GRS=Genetic risk score, GRS:AP=Interaction between GRS and AP, NO_2_=nitrogen dioxide, NO_x_=nitrogen oxides, PM_2.5/10/coarse_=particulate matter with median aerodynamic diameters of ≤2.5/≤10/ 2.5–10 μm, PM_2.5 absorbance=_ the reflectance of PM_2.5_ filters,*=P<0.05 | | | | | | |

**Table S11: Description of the study subgroups according to healthy or unhealthy housing conditions.**

|  | **Healthy** | **Unhealthy** |
| --- | --- | --- |
| N | 171 | 387 |
| Chronic lung diseases No. (%) | 43 (25.1) | 113 (29.2) |
| Mean age [years] ± SD | 73.5 ± 2.9 | 73.5 ± 3.0 |
| Mean Body Mass Index [kg/m²] ± SD | 27.3 ± 4.5 | 27.3 ± 4.2 |
| Educational level of the participant or spouse No. (%): | | |
| Less than 10 years | 30 (17.5) | 66 (17.1) |
| 10 years | 88 (51.5) | 189 (49.0) |
| More than 10 years | 53 (31.0) | 131 (33.9) |
| Smoking No. (%): | | |
| ever smoker | 19 (11.1) | 84 (21.7) |
| passive smoking | 0 (0.0) | 341 (88.3) |
| Indoor air pollution No. (%) | 0 (0.0) | 138 (35.7) |
| Residential move No. (%) | 18 (10.5) | 53 (13.7) |
| Median chronic air pollution exposure (IQR): | | |
| NO_2_ [µg/m^3^] | 28.2 [8.4] | 30.6 [12.9] |
| NO_x_ [µg/m^3^] | 44.1 [23.2] | 51.7 [33.9] |
| PM_2.5_ [µg/m^3^] | 24.8 [2.6] | 25.8 [3.4] |
| PM_10_ [µg/m^3^] | 37.5 [3.4] | 38.6 [4.3] |
| PM_coarse_ [µg/m^3^] | 12.7 [2.6] | 13.4 [3.5] |
| PM_2.5 absorbance_ [10^-5^/m] | 1.8 [0.6] | 2.0 [0.7] |
| Individuals exposed to air pollution higher than the European Union threshold No. (%) | 74 (46.8) | 218 (59.9) |
| Healthy housing conditions= no dampness, mould, cooking with gas, or passive smoking exposure at home  Chronic lung diseases= any of the following conditions: asthma, chronic bronchitis, cough (with sputum), or chronic obstructive pulmonary disease ever diagnosed by a physician at the second follow-up examination  Chronic air pollution exposure of 15 years before the respiratory assessments= average of annual mean exposures at the baseline and first follow-up examinations  European Union threshold=NO_2_: 40 µg/m^3^; PM_2.5_: 25 µg/m^3^; PM_10_: 40 µg/m^3 28^  IQR=interquartile ranges, NO_2_=nitrogen dioxide, NO_x_=nitrogen oxides, PM_2.5/10/coarse_=particulate matter with median aerodynamic diameters of ≤2.5/≤10/ 2.5–10 μm, PM_2.5 absorbance=_ the reflectance of PM_2.5_ filters, SD=standard deviation | | |

**Table S12: Adjusted gene-environment interaction effects on chronic lung diseases in elderly women living in healthy housing conditions.**

| **Air Pollutant** | **Term** | **Odds Ratio** | **Odds Ratio 95% CI** | **beta** | **beta 95% CI** | **P** |
| --- | --- | --- | --- | --- | --- | --- |
| NO_2_ | GRS | 0.751 | 0.445;1.267 | -0.287 | -0.810;0.237 | 0.283 |
| NO_2_ | AP | 1.717 | 0.909;3.243 | 0.541 | -0.095;1.177 | 0.096 |
| NO_2_ | GRS:AP | 0.442 | 0.164;1.191 | -0.817 | -1.808;0.175 | 0.107 |
| NO_x_ | GRS | 0.763 | 0.453;1.285 | -0.270 | -0.791;0.251 | 0.310 |
| NO_x_ | AP | 1.797 | 0.965;3.343 | 0.586 | -0.035;1.207 | 0.064 |
| NO_x_ | GRS:AP | 0.529 | 0.199;1.412 | -0.636 | -1.617;0.345 | 0.204 |
| PM_2.5_ | GRS | 0.850 | 0.516;1.400 | -0.163 | -0.662;0.336 | 0.523 |
| PM_2.5_ | AP | 1.516 | 0.873;2.631 | 0.416 | -0.136;0.967 | 0.140 |
| PM_2.5_ | GRS:AP | 0.742 | 0.346;1.593 | -0.298 | -1.061;0.466 | 0.445 |
| PM_10_ | GRS | 0.815 | 0.492;1.349 | -0.205 | -0.709;0.299 | 0.426 |
| PM_10_ | AP | 1.571 | 0.928;2.659 | 0.452 | -0.074;0.978 | 0.092 |
| PM_10_ | GRS:AP | 0.786 | 0.377;1.636 | -0.241 | -0.975;0.492 | 0.519 |
| PM_2.5 absorbance_ | GRS | 0.795 | 0.474;1.332 | -0.230 | -0.746;0.287 | 0.383 |
| PM_2.5 absorbance_ | AP | 1.554 | 0.956;2.527 | 0.441 | -0.045;0.927 | 0.076 |
| PM_2.5 absorbance_ | GRS:AP | 0.642 | 0.272;1.517 | -0.443 | -1.303;0.417 | 0.313 |
| PM_coarse_ | GRS | 0.809 | 0.482;1.356 | -0.212 | -0.729;0.304 | 0.420 |
| PM_coarse_ | AP | 1.532 | 0.838;2.803 | 0.427 | -0.177;1.031 | 0.166 |
| PM_coarse_ | GRS:AP | 0.453 | 0.187;1.093 | -0.793 | -1.675;0.089 | 0.078 |
| Healthy housing conditions= no dampness, mould, cooking with gas, or passive smoking exposure at home,  Effect estimates per one IQR increase in GRS or air pollution exposure adjusted for age, body mass index, educational level (low as the reference, medium, high), smoking status (never as the reference, ever), passive smoking (never as the reference, ever), indoor air pollution (dampness, mould or cooking with gas at home), and residential moving in the observation time. N=171 observations for GRS construction, N=158 observations with complete data for GxE interaction testing.  AP=Air pollutant, CI=confidence intervals, GRS=Genetic risk score, GRS:AP=Interaction between GRS and AP, NO_2_=nitrogen dioxide, NO_x_=nitrogen oxides, PM_2.5/10/coarse_=particulate matter with median aerodynamic diameters of ≤2.5/≤10/ 2.5–10 μm, PM_2.5 absorbance=_ the reflectance of PM_2.5_ filters,*=P<0.05 | | | | | | |

**Table S13: Adjusted gene-environment interaction effects on chronic lung diseases in elderly women living in unhealthy housing conditions.**

| **Air Pollutant** | **Term** | **Odds Ratio** | **Odds Ratio 95% CI** | **beta** | **beta 95% CI** | **P** |
| --- | --- | --- | --- | --- | --- | --- |
| NO_2_ | GRS | 1.191 | 0.861;1.647 | 0.175 | -0.149;0.499 | 0.290 |
| NO_2_ | AP | 1.611 | 1.203;2.156 | 0.477 | 0.185;0.768 | 0.001* |
| NO_2_ | GRS:AP | 0.604 | 0.402;0.907 | -0.505 | -0.912;-0.097 | 0.015* |
| NO_x_ | GRS | 1.191 | 0.863;1.645 | 0.175 | -0.148;0.498 | 0.288 |
| NO_x_ | AP | 1.470 | 1.107;1.952 | 0.385 | 0.101;0.669 | 0.008* |
| NO_x_ | GRS:AP | 0.564 | 0.368;0.863 | -0.573 | -0.998;-0.148 | 0.008* |
| PM_2.5_ | GRS | 1.249 | 0.895;1.742 | 0.222 | -0.110;0.555 | 0.190 |
| PM_2.5_ | AP | 1.747 | 1.261;2.422 | 0.558 | 0.232;0.884 | 0.001* |
| PM_2.5_ | GRS:AP | 0.572 | 0.385;0.848 | -0.559 | -0.953;-0.165 | 0.005* |
| PM_10_ | GRS | 1.192 | 0.864;1.644 | 0.176 | -0.146;0.497 | 0.285 |
| PM_10_ | AP | 1.521 | 1.123;2.058 | 0.419 | 0.116;0.722 | 0.007* |
| PM_10_ | GRS:AP | 0.680 | 0.484;0.955 | -0.386 | -0.726;-0.046 | 0.026* |
| PM_2.5 absorbance_ | GRS | 1.185 | 0.859;1.634 | 0.170 | -0.152;0.491 | 0.302 |
| PM_2.5 absorbance_ | AP | 1.535 | 1.188;1.984 | 0.429 | 0.172;0.685 | 0.001* |
| PM_2.5 absorbance_ | GRS:AP | 0.720 | 0.523;0.990 | -0.329 | -0.648;-0.010 | 0.043* |
| PM_coarse_ | GRS | 1.183 | 0.856;1.634 | 0.168 | -0.155;0.491 | 0.309 |
| PM_coarse_ | AP | 1.761 | 1.257;2.467 | 0.566 | 0.229;0.903 | 0.001* |
| PM_coarse_ | GRS:AP | 0.657 | 0.434;0.994 | -0.420 | -0.835;-0.006 | 0.047* |
| Unhealthy housing conditions= dampness, mould, cooking with gas, or passive smoking exposure at home,  Effect estimates per one IQR increase in GRS or air pollution exposure adjusted for age, body mass index, educational level (low as the reference, medium, high), smoking status (never as the reference, ever), passive smoking (never as the reference, ever), indoor air pollution (dampness, mould or cooking with gas at home), and residential moving in the observation time. N=387 observations for GRS construction, N=362 observations with complete data for GxE interaction testing.  AP=Air pollutant, CI=confidence intervals, GRS=Genetic risk score, GRS:AP=Interaction between GRS and AP, NO_2_=nitrogen dioxide, NO_x_=nitrogen oxides, PM_2.5/10/coarse_=particulate matter with median aerodynamic diameters of ≤2.5/≤10/ 2.5–10 μm, PM_2.5 absorbance=_ the reflectance of PM_2.5_ filters,*=P<0.05 | | | | | | |

**References**

1. Broer L, Buchman AS, Deelen J *et al.* GWAS of Longevity in CHARGE Consortium Confirms APOE and FOXO3 Candidacy. *The Journals of Gerontology: Series A* 2015;**70**:110–8.

2. Joshi PK, Pirastu N, Kentistou KA *et al.* Genome-wide meta-analysis associates HLA-DQA1/DRB1 and LPA and lifestyle factors with human longevity. *Nat Commun* 2017;**8**:910.

3. Newman AB, Walter S, Lunetta KL *et al.* A meta-analysis of four genome-wide association studies of survival to age 90 years or older: the Cohorts for Heart and Aging Research in Genomic Epidemiology Consortium. *The Journals of Gerontology: Series A* 2010;**65**:478–87.

4. Pilling LC, Kuo C-L, Sicinski K *et al.* Human longevity: 25 genetic loci associated in 389,166 UK biobank participants. *Aging (Albany NY)* 2017;**9**:2504–20.

5. Partridge L, Deelen J, Slagboom PE. Facing up to the global challenges of ageing. *Nature* 2018;**561**:45–56.

6. Singh PP, Demmitt BA, Nath RD, Brunet A. The Genetics of Aging: A Vertebrate Perspective. *Cell* 2019;**177**:200–20.

7. Edwards DRV, Gilbert JR, Hicks JE *et al.* Linkage and association of successful aging to the 6q25 region in large Amish kindreds. *Age (Dordr)* 2013;**35**:1467–77.

8. Walter S, Atzmon G, Demerath EW *et al.* A genome-wide association study of aging. *Neurobiol Aging* 2011;**32**:2109.e15-28.

9. Atkins JL, Jylhävä J, Pedersen NL *et al.* A genome-wide association study of the frailty index highlights brain pathways in ageing. *Aging Cell* 2021;**20**:e13459.

10. Yashin AI, Wu D, Arbeeva LS *et al.* Genetics of aging, health, and survival: dynamic regulation of human longevity related traits. *Front Genet* 2015;**6**:122.

11. Poduslo SE, Huang R, Spiro A. A genome screen of successful aging without cognitive decline identifies LRP1B by haplotype analysis. *Am J Med Genet B Neuropsychiatr Genet* 2010;**153B**:114–9.

12. Le Clerc S, Taing L, Ezzedine K *et al.* A genome-wide association study in Caucasian women points out a putative role of the STXBP5L gene in facial photoaging. *J Invest Dermatol* 2013;**133**:929–35.

13. Lunetta KL, D'Agostino RB, Karasik D *et al.* Genetic correlates of longevity and selected age-related phenotypes: a genome-wide association study in the Framingham Study. *BMC Med Genet* 2007;**8**:S13.

14. Deelen J, Beekman M, Uh H-W *et al.* Genome-wide association meta-analysis of human longevity identifies a novel locus conferring survival beyond 90 years of age. *Hum Mol Genet* 2014;**23**:4420–32.

15. Malovini A, Illario M, Iaccarino G *et al.* Association study on long-living individuals from Southern Italy identifies rs10491334 in the CAMKIV gene that regulates survival proteins. *Rejuvenation Res* 2011;**14**:283–91.

16. Flachsbart F, Ellinghaus D, Gentschew L *et al.* Immunochip analysis identifies association of the RAD50/IL13 region with human longevity. *Aging Cell* 2016;**15**:585–8.

17. Willcox BJ, Donlon TA, He Q *et al.* FOXO3A genotype is strongly associated with human longevity. *Proc Natl Acad Sci U S A* 2008;**105**:13987–92.

18. Ji JS, Liu L, Yan LL, Zeng Y. Comparing Effects of FOXO3 and Residing in Urban Areas on Longevity: A Gene-Environment Interaction Study. *The Journals of Gerontology: Series A* 2022;**77**:1549–56.

19. Zeng Y, Nie C, Min J *et al.* Novel loci and pathways significantly associated with longevity. *Sci Rep* 2016;**6**:21243.

20. Sebastiani P, Gurinovich A, Bae H *et al.* Four Genome-Wide Association Studies Identify New Extreme Longevity Variants. *The Journals of Gerontology: Series A* 2017;**72**:1453–64.

21. Emanuele E, Fontana JM, Minoretti P, Geroldi D. Preliminary evidence of a genetic association between chromosome 9p21.3 and human longevity. *Rejuvenation Res* 2010;**13**:23–6.

22. Fortney K, Dobriban E, Garagnani P *et al.* Genome-Wide Scan Informed by Age-Related Disease Identifies Loci for Exceptional Human Longevity. *PLoS Genet* 2015;**11**:e1005728.

23. Yao Y, Liu L, Guo G, Zeng Y, Ji JS. Interaction of Sirtuin 1 (SIRT1) candidate longevity gene and particulate matter (PM2.5) on all-cause mortality: a longitudinal cohort study in China. *Environ Health* 2021;**20**:25.

24. Nebel A, Kleindorp R, Caliebe A *et al.* A genome-wide association study confirms APOE as the major gene influencing survival in long-lived individuals. *Mech Ageing Dev* 2011;**132**:324–30.

25. Sollis E, Mosaku A, Abid A *et al.* The NHGRI-EBI GWAS Catalog: knowledgebase and deposition resource. *Nucleic Acids Res* 2023;**51**:D977-D985.

26. Hüls A, Ickstadt K, Schikowski T, Krämer U. Detection of gene-environment interactions in the presence of linkage disequilibrium and noise by using genetic risk scores with internal weights from elastic net regression. *BMC Genet* 2017;**18**:55.

27. Ottman R. Gene-environment interaction: definitions and study designs. *Prev Med* 1996;**25**:764–70.

28. The European Parliament and the Council. The European Parliament and the Council. Directive 2008/50/EC of the European Parliament and of the Council of 21 May 2008 on ambient air quality and cleaner air for Europe: RL 2008/50/EG, 2008.
